# Correspondence between BOLD fMRI task response and cerebrovascular reactivity across the cerebral cortex

**DOI:** 10.3389/fphys.2023.1167148

**Published:** 2023-05-09

**Authors:** Rebecca J. Williams, Jacinta L. Specht, Erin L. Mazerolle, R. Marc Lebel, M. Ethan MacDonald, G. Bruce Pike

**Affiliations:** ^1^ Faculty of Health, School of Human Services, Charles Darwin University, Darwin, NT, Australia; ^2^ Department of Clinical Neuroscience, Cumming School of Medicine, University of Calgary, Calgary, AB, Canada; ^3^ Department of Radiology, Cumming School of Medicine, University of Calgary, Calgary, AB, Canada; ^4^ Hotchkiss Brain Institute, Cumming School of Medicine, University of Calgary, Calgary, AB, Canada; ^5^ Departments of Psychology and Computer Science, St. Francis Xavier University, Antigonish, NS, Canada; ^6^ GE HealthCare, Calgary, AB, Canada; ^7^ Department of Biomedical Engineering, Schulich School of Engineering, University of Calgary, Calgary, AB, Canada; ^8^ Department of Electrical and Software Engineering, Schulich School of Engineering, University of Calgary, Calgary, AB, Canada

**Keywords:** BOLD, cerebrovascular reactivity, hypercapnia, functional magnet resonance imaging (fMRI), cerebral blood blow, vascular physiology, attention, cognition

## Abstract

BOLD sensitivity to baseline perfusion and blood volume is a well-acknowledged fMRI confound. Vascular correction techniques based on cerebrovascular reactivity (CVR) might reduce variance due to baseline cerebral blood volume, however this is predicated on an invariant linear relationship between CVR and BOLD signal magnitude. Cognitive paradigms have relatively low signal, high variance and involve spatially heterogenous cortical regions; it is therefore unclear whether the BOLD response magnitude to complex paradigms can be predicted by CVR. The feasibility of predicting BOLD signal magnitude from CVR was explored in the present work across two experiments using different CVR approaches. The first utilized a large database containing breath-hold BOLD responses and 3 different cognitive tasks. The second experiment, in an independent sample, calculated CVR using the delivery of a fixed concentration of carbon dioxide and a different cognitive task. An atlas-based regression approach was implemented for both experiments to evaluate the shared variance between task-invoked BOLD responses and CVR across the cerebral cortex. Both experiments found significant relationships between CVR and task-based BOLD magnitude, with activation in the right cuneus (*R* ^2^ = 0.64) and paracentral gyrus (*R* ^2^ = 0.71), and the left pars opercularis (*R* ^2^ = 0.67), superior frontal gyrus (*R* ^2^ = 0.62) and inferior parietal cortex (*R* ^2^ = 0.63) strongly predicted by CVR. The parietal regions bilaterally were highly consistent, with linear regressions significant in these regions for all four tasks. Group analyses showed that CVR correction increased BOLD sensitivity. Overall, this work suggests that BOLD signal response magnitudes to cognitive tasks are predicted by CVR across different regions of the cerebral cortex, providing support for the use of correction based on baseline vascular physiology.

## 1 Introduction

Functional MRI (fMRI) based on blood oxygen level-dependent (BOLD) contrast is widely used in neuroscience research and represents a composite signal arising from changes in total intravoxel deoxyhemoglobin (Kim and Ogawa, 2012). Transient reductions in deoxyhemoglobin due to changes in cerebral blood flow, blood volume and cerebral metabolic of oxygen give rise to BOLD signal changes capitalized by both task-based and resting-state fMRI to probe neural networks (Hoge et al., 1999; Pike, 2012). These dynamic changes are highly dependent on basal physiology, with resting perfusion, blood volume and venous oxygenation having strong modulatory effects on the BOLD signal (Stefanovic et al., 2006; Liu et al., 2013a; Chu et al., 2018). The negative consequences of this includes increased inter-subject variation and the problematic interpretation of BOLD fMRI signal changes in situations where independent basal perfusion changes occur, such as in aging (Chen et al., 2011; Chen, 2019; MacDonald et al., 2020; Juttukonda et al., 2021) and cerebrovascular disease (Blicher et al., 2012; MacDonald et al., 2016; Williams et al., 2017; Mazerolle et al., 2018). Scaling techniques have been proposed to address BOLD signal magnitude differences due to basal physiology (Biswal et al., 2007; Kannurpatti and Biswal, 2008; Tsvetanov et al., 2015; Kazan et al., 2016; Tsvetanov et al., 2021b). Hypercapnic normalization is a scaling technique where measurements of cerebrovascular reactivity (CVR) are implemented to reduce fMRI activation map dependence on baseline cerebral blood volume (Bandettini and Wong, 1997; Cohen et al., 2004; Liau and Liu, 2009). CVR refers to the ability of blood vessels to dynamically regulate cerebral blood flow and is often measured with MRI in the assessment of vascular health (Pillai and Mikulis, 2015; Chen, 2018). While CVR is traditionally defined as the change in flow due to vasoactive stimulus (Fisher and Mikulis, 2021), BOLD contrast is often used as a surrogate measure of cerebral blood flow with the assumption of the vasoactive stimulus being isometabolic (Chen and Pike, 2010).

There are different vasoactive stimuli that can be applied to measure CVR, the two most common being acetazolamide and carbon dioxide (CO_2_). Breath-hold induced hypercapnia and the delivery of air mixtures with increased concentrations of CO_2_ are the most commonly used approaches in MRI research studies. Participants holding their breath for short (10–30 s) epochs is a relatively easy and reliable technique for inducing mild hypercapnia (Bright and Murphy, 2013; Pinto et al., 2020). Breath-hold based measures of CVR have also shown good correspondence with those obtained with respiratory manipulation via the delivery of gas mixtures containing elevated concentrations of CO_2_ (Tancredi and Hoge, 2013). This latter approach involves both the delivery of gases and the precise recording of end-tidal gases and has been shown to have good test-retest reliability in non-patient groups (Liu et al., 2021).

Blood flow modulations underpinning BOLD responses to neural activity are caused by neurovascular coupling, where changes in regional flow reflect modulations in neural activity and metabolic demand (Hosford and Gourine, 2019). This is independent from the mechanisms related to acidosis that regulate cerebral blood flow increases under hypercapnic conditions (Battisti-Charbonney et al., 2011; Duffin et al., 2021). Despite these different pathways leading to hyperemia, contributions from mutual physiology such as baseline cerebral blood volume suggests that CVR may be a BOLD signal modulator. This is critical for the implementation of hypercapnic normalization, which is predicated on a linear relationship between CVR and neural activity-induced BOLD signal changes. There is some evidence supporting this relationship. For instance, when investigating how different baseline measures of vascular physiology modulate task-induced BOLD signals, Liu et al. showed a correspondence between BOLD signal magnitude to a visual scene-categorization task and CVR in four different regions-of-interest (ROIs) including the early visual areas, medial temporal lobe, and bilateral inferior frontal gyrus (Liu et al., 2013a). Another study characterised the relationship between breath-hold BOLD and task-BOLD signal changes in younger and older adults (Kannurpatti et al., 2014). The tasks included a motor and a cognitive task associated with executive control function. When evaluating the relationship between task and breath-hold BOLD responses in significantly activated voxels, the authors found a linear relationship for the younger group only (Kannurpatti et al., 2014). Addressing discrepancies in CVR and task-based BOLD responses between younger and older adults was the aim of research by Liu et al. (2013b). In this study, hypercapnic normalization was implemented where CVR maps were calculated on a per-subject basis to normalize BOLD activation maps to a memory task in older and younger adults. Age-related decreases in BOLD signal were found primarily in the posterior visual cortex and temporal lobe; however, these decreases were no longer observed following normalization. This study suggested that vascular-driven age-related differences in BOLD activation to a memory task can be addressed using hypercapnic normalization.

These findings highlight that one of the potential benefits of adding CVR to a task-based fMRI experiment is to correct age-related differences in BOLD responses due to baseline vasculature. This benefit would extend to all populations in group fMRI studies by reducing inter-subject variability attributable to baseline vasculature. Hypercapnic normalization has been shown to reduce variability using breath-hold CVR in young adults (Thomason et al., 2007), and it can be concluded from the literature, summarized above, that activation magnitude can be predicted by CVR for a small number of tasks in some regions of cortex. This suggests that CVR can be incorporated into a linear model to account for variance and reduce error. An important consideration however is that these conclusions are based on a small number of studies that are limited in both sample sizes and the tasks evaluated. An under-explored question is whether vascular correction is applicable for all fMRI paradigms and brain regions. Cortical regions subserving higher-order cognitive processes show more inter-subject variability than primary sensory cortices in both structure (Sydnor et al., 2021) and function (Mueller et al., 2013). Spatial disparity has also been observed in blood flow and metabolic coupling (Chiarelli et al., 2007), underscoring the importance of characterizing the relationship between vascular physiology and neural-activity mediated BOLD signal on a regional level. Relative to sensory tasks targeting the unimodality regions such as the primary visual or motor cortices, cognitive fMRI tasks have lower sensitivity due to the use of multiple experimental conditions and cognitive subtraction (Logothetis, 2008). It has been suggested that cognitive paradigms are dominated by neural response variability, and vascular correction might therefore be ineffective (Kannurpatti et al., 2010). Further work investigating the utility of vascular correction for cognitive paradigms is warranted. Furthermore, all studies to date have implemented only one type of CVR technique; whether the hypercapnic approach affects the relationship between CVR and task-related BOLD signal remains undetermined.

The aim of the present research was to thoroughly investigate the relationship between CVR and BOLD responses to cognitive tasks implicating different neural networks. Two experimental studies were implemented to meet this aim, each utilizing different approaches to measure CVR using hypercapnia: breath-hold and gas inhalation. It was hypothesized that neural-activity mediated BOLD responses would be linearly related to CVR across the cerebral cortex for both hypercapnic approaches. The first experiment involved the analysis of an open-source dataset containing breath-hold and task-based BOLD fMRI data from the UCLA Consortium for Neuropsychiatric Phenomics (CNP) LA5c Study (Poldrack et al., 2016). In this first experiment, the linear relationship between CVR, inferred using breath-hold BOLD responses, and task-based BOLD responses across different cognitive networks was measured. Group analyses were performed to determine if vascular scaling using breath-hold BOLD responses reduces variability in large datasets. The second experiment attempted to determine if the results from the first experiment generalize to gas-inhalation CVR. This was achieved by characterizing the relationship between CVR and BOLD activation to an attention task in an independent sample with CO_2_-administered hypercapnia.

## 2 Methods and materials

### 2.1 Experiment 1: Breath-hold CVR

#### 2.1.1 Participants

For study 1, all data were obtained from the OpenfMRI database (Bilder et al., 2020). Data were downloaded from https://openneuro.org/datasets/ds000030/versions/1.0.0. Full details regarding participant recruitment, inclusion and exclusion criteria are provided in the study protocol paper (Poldrack et al., 2016). All participants gave written informed consent, and the study was approved by the Institutional Review Board at UCLA. The complete dataset contains images from healthy control subjects, and those diagnosed with schizophrenia, bipolar disorder, and attention deficit/hyperactivity disorder. Only the data from control subjects were utilised in this work. All control participants with task-based fMRI including the 3 paradigms utilized in the present study (detailed in task descriptions below) and T_1_-weighted structural data were included in the present analysis, resulting in a total of 114 participants (mean age = 31.46 ± 8.78 years, range = 21–50 years, 54 F).

#### 2.1.2 Imaging acquisition

All MRI data for Study 1 were acquired on one of two 3 T Siemens Trio scanners. Subjects completed two separate scanning sessions in a counterbalanced order. The structural and fMRI data used in the present analysis were acquired in different sessions. An MPRAGE was acquired in the sagittal orientation (TR/TE/TI = 1900/2.26/1,100 ms, FOV = 250 mm, matrix = 256 × 256, flip angle = 7°, slice thickness = 1 mm, 176 slices). The fMRI data (cognitive tasks and breath-hold) were acquired using T_2_*-weighted echo planar imaging with TR/TE = 2000/30 ms, flip angle = 90°, FOV = 192 mm, matrix = 64 × 64, slice thickness = 4 mm, 34 slices.

#### 2.1.3 Task descriptions

Study participants completed a battery of cognitive paradigms during the fMRI scans, including three event-related cognitive tasks and one breath-holding task. While more than three cognitive tasks were available, not every participant completed the full battery. The following three cognitive tasks were specifically chosen for this analysis because they were completed by the most participants, resulting in the final sample size of 114. A detailed description of the tasks is given in (Poldrack et al., 2016).

The first of the three cognitive tasks, the Spatial Capacity Task (SCAP), targeted spatial working memory (Glahn et al., 2003), where stimuli included pseudo-randomly displayed yellow circles (1, 3, 5 or 7) around a central fixation cross, presented for 2 s per trial. After a short delay (1.5, 3 or 4.5 s), a single green circle appeared. The participant was required to indicate whether the green circle was in the same position as one of the yellow target circles. A total of 48 trials were presented. The manipulated variables were number of target circles, with 4 levels (1, 3, 5, 7) and delay in seconds (1.5, 3 or 4.5).

The second task was the stop-signal task, a measure of response inhibition (Logan et al., 1984). The participants were instructed to press a button as quickly as possible in response to a presented ‘go’ stimulus (arrows pointing to the left or to the right). In 25% of trials, the arrow stimulus was presented with an auditory tone (presented through headphones for 250 ms), which indicated a ‘stop’ signal. Participants were instructed to withhold all button presses for trials with the ‘stop’ signal. There was a total of 128 trials, of which 32 were stop trials. Trials were 1,000 ms in duration and interspersed with a jittered baseline period consisting of a fixation cross.

The third cognitive task was a task-switching paradigm (Miyake et al., 2004). For each trial, one of four stimuli including a red triangle, red circle, green triangle, or green circle was presented. Participants made a button-press response as quickly as possible in response to the stimulus to indicate the color (red or green) or shape (triangle or circle). A cue presented directly prior to the onset of the stimulus instructed the participant to respond to either the color or the shape of the stimulus. This cue was either the full word (‘Color’ or ‘Shape’), or the first letter (‘C’ or ‘S’). A total of 96 trials were presented, with 33% of all trials involving a switch of instructions compared to prior trials.

To measure CVR, a breath-hold task was performed. Participants held their breath for periods of 13.5 s, interspersed with 16.5 s of regular breathing. A visual cue was presented to prepare and pace the breath-holding challenge. There were 5 epochs of breath-holding across a total of 2.5 min. A respiratory belt was worn by participants during the breath-hold task, although these data was not analyzed in this analysis.

#### 2.1.4 fMRI preprocessing

**FIGURE 1:**
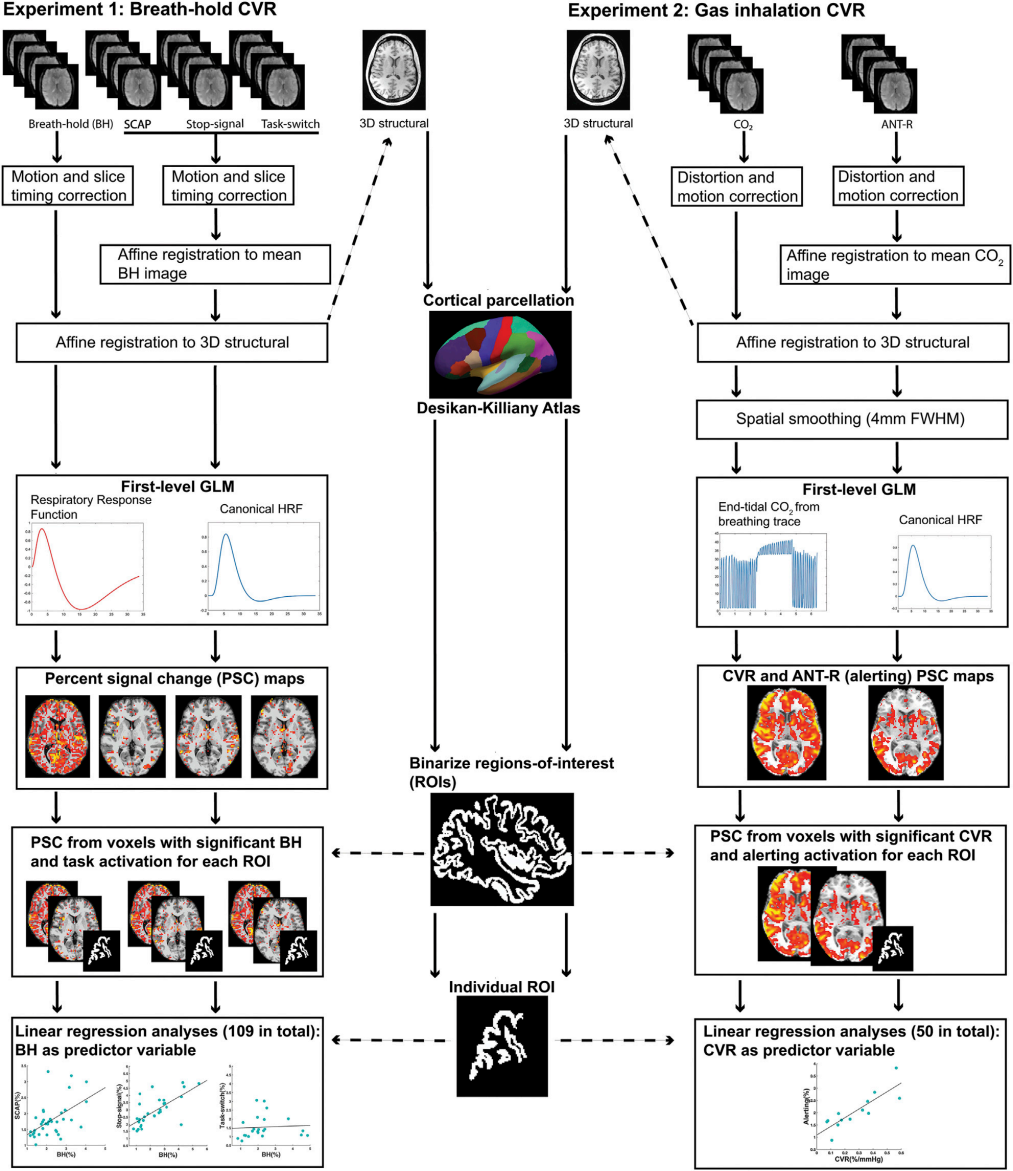
Schematic diagram summarizing the pre-processing and first-level analysis pipeline for experiment 1 (left) and experiment 2 (right).

A schematic summarizing the pre-processing pipeline for experiment 1 is shown in Figure 1. All fMRI data underwent slice timing correction and motion correction using SPM12 (https://www.fil.ion.ucl.ac.uk/spm/). To ensure precise registration between breath-hold and cognitive tasks, for each subject all fMRIs were aligned using affine registration with Advanced Normalization Tools (Avants et al., 2011; Avants et al., 2014). Affine registration between each subject’s fMRIs and structural T_1_ MPRAGE, and normalisation of the T_1_ image to the MNI template, was performed using Unified Segmentation in SPM12 (Ashburner and Friston, 2005). Deformation fields from the T_1_ image segmentation were used to normalize the fMRIs to the MNI template. No spatial smoothing was performed.

#### 2.1.5 fMRI first-level analyses

The fMRI data corresponding to the three tasks were individually entered into first-level general linear models. Modelling of the SCAP task included separate regressors for all 4 levels of load (1, 3, 5, 7) at each delay time (1.5, 3, 5 s) resulting in 12 task-related regressors. The contrast of interest was load 5 (averaged over all delays) relative to baseline, as this load has been implemented previously to interrogate working memory within capacity limits (Todd and Marois, 2004; Montojo et al., 2014). The model for the stop-signal task included 4 task-related regressors: successful go, failed go, successful stop, and failed stop. The contrast of interest was successful stop. The model for the task-switching task included individual regressors for the 4 types of cue (‘SHAPE’, ‘S’, ‘COLOR’, ‘C’) and whether each cue was a switch or a no-switch trial, resulting in a total of 8 task-related regressors. The contrast of interest was the effect of the switch trials averaged over all cues. For all 3 cognitive paradigms, the task-related regressors were modelled using delta functions corresponding to the onset times of the trials, convolved with the canonical hemodynamic response function. Only correct responses were included in the task-related regressors, with incorrect responses modelled as separate regressors of no interest. Each model also included six motion parameters to account for variability due to head motion.

The BOLD responses to the breath-hold epochs were modelled using a ramp function convolved with a respiratory response function (Birn et al., 2008). A ramp function was used instead of a typical boxcar approach to model the accumulation of CO_2_ over the course of the breath-hold epoch. The respiratory response function was established by Birn et al. (2008) to accurately model hypercapnia-induced BOLD responses to breath-hold. Similar to prior work modelling the hemodynamic response function (Handwerker et al., 2004; Williams et al., 2014; Williams et al., 2016), the respiratory version is modelled by the difference between two gamma functions but characterised by a longer, larger post-stimulus under-shoot. Both response functions can both be observed in Figure 1. For a small subset of participants (15/114, or 13%), the respiratory response function was not a good fit and resulted in too few significant voxels available for the ROI analysis. Rather than exclude these participants from further analysis, the hemodynamic response function convolved with the ramp function was implemented instead as this was a better fit for their breath-hold data. To account for possible delays in BOLD responses to breath-hold induced hypercapnia, different general linear models were created for each participant with staggered onset times. The onset times increased in increments of 1 TR (2 s), up to a maximum of 12 s. The model with the most significant voxels for each individual subject was utilized for further analyses. The contrast of interest used in further analyses was the effect of the breath-hold epochs greater than baseline.

#### 2.1.6 ROI analysis

Structural ROIs were obtained on a per-subject basis using each participant’s T_1_-weighted image and cortical reconstruction with the recon-all function in FreeSurfer image analysis suite (Dale et al., 1999; Fischl et al., 1999; Fischl et al., 2004). This included automatic labelling of all cortical regions based on the Desikan-Killiany Atlas (Desikan et al., 2006). This atlas parcellates each of the cerebral hemispheres into 34 distinct regions based on gyral anatomy, resulting in 68 anatomical ROIs per subject.

To perform the ROI analysis, BOLD voxel-wise percent signal change (PSC) values for each of the cognitive and breath-hold tasks were extracted from each anatomical ROI on a per-subject basis. The following procedure was performed on individual activation maps from the first-level analysis. First, PSC maps were calculated for each task using the contrast images corresponding to the contrast of interest (outlined in the ‘fMRI first-level analyses’ section above) and the baseline images. The PSC maps were thresholded so that only significant (*p* < 0.001 uncorrected for multiple comparisons) voxels were included in the ROI analyses.

Once the thresholded PSC maps were calculated for all 4 fMRI tasks (3 cognitive tasks and breath-hold), each of the anatomical ROIs were binarized and transformed into fMRI space. The voxel values of the thresholded PSC maps were then extracted for each anatomical ROI. For each of the 3 cognitive tasks, only voxels with both significant task activation and a significant BOLD response to the breath-hold task were included. The voxels values were then averaged for each ROI, resulting in 6 averaged ROI PSC values for each participant: one for each of the 3 cognitive tasks, and its corresponding breath-hold. Any individuals with breath-hold ROI values that were considered outliers for the ROI were removed from the analysis in order to minimize effects from large vessels. Outliers were determined as values greater than 3 mean absolute deviations. If fewer than 10 participants demonstrated significant activation within an ROI, that ROI was removed from the analysis. Prior research showing that a sample size of 8 is sufficient for regression models with little variance, while a minimum of 25 is required for models with high variance (Jenkins and Quintana-Ascencio, 2020). For the present research it was anticipated that variance would differ between ROIs, and choosing too high a threshold in terms of number of subjects with significant activation might eliminate important results.

For each cognitive task, linear regression analyses were run for each ROI PSC, with the breath-hold PSC as the predictor variable. This resulted in a possible 204 regressions (68 ROIs x 3 cognitive tasks), although not all participants showed significant activation in all ROIs and therefore a subset was excluded. The final number of regression analyses performed across all 3 tasks was 109. Outliers were removed prior to regression analyses, with outliers defined as data points greater than 3 standard deviations from the group mean. All linear regression analyses were run in MATLAB. Due to inflated probability of making a Type I error due to the number of linear regressions performed, False Discovery Rate (FDR) correction (*p* < 0.05) was implemented.

#### 2.1.7 Group-level analyses

The aim of the group-level analyses was to determine whether correcting for CVR by including breath-hold covariates into the general linear model reduced variance and increased sensitivity. The inclusion of covariates has shown to be a robust method to correct for CVR (Liau and Liu, 2009), and can be achieved either at the voxel-wise or the ROI level. Both voxel and ROI methods were explored here. For the voxel-wise approach, the breath-hold contrast image for each subject was entered into the model as a covariate. This was achieved using the extended version (Yang et al., 2011) of the Biological Parametric Mapping toolbox (Casanova et al., 2007), an SPM toolbox which allows for image covariates by implementing a separate general linear model for each voxel. CVR uncorrected analyses for the voxel-wise method were standard group-level one-sample *t*-test performed in SPM12 for each task separately. All *t*-maps output were thresholded at *p* < 0.05 cluster-corrected for multiple comparisons using AFNI functions 3dFWHMx and 3dClustSim. For the ROI method, regions were chosen from the significant ROIs from the linear regression analyses (outlined in section 2.1.6). The averaged breath-hold value for each ROI was entered for each individual subject into the model as a covariate, and the statistical search limited to the ROI using binary masks. The CVR uncorrected analyses for the ROI method were identical except no covariate was included into the model. This was achieved using standard second-level analyses in SPM12.

### 2.2 Experiment 2: Gas inhalation CVR

#### 2.2.1 Participants

For the second study, 17 participants completed a single scan session consisting of a cognitive fMRI scan, a hypercapnic fMRI scan with delivered CO_2_ gas mixtures (see task and CO_2_ paradigm descriptions below), and a structural scan. All participants reported no history of neurologic or respiratory disease. Data from 2 participants were removed due to excessive head motion and incomplete CO_2_ scan. The final analysis presented here consisted of 15 participants (mean age = 38.5 ± 13.5 years, age range = 22–58 years, 8 F, all right-handed). This study was approved by the University of Calgary Conjoint Health Research Ethics Board and all participants provided written informed consent.

#### 2.2.2 Imaging acquisition

All images for study 2 were acquired on a 3 T GE MRI scanner (Discovery MR750) with a 32-channel head coil from Nova Medical. The fMRI acquisition consisted of T_2_*-weighted echo planar images with multiband (MB) acceleration (MB factor = 3), TR/TE = 1800/30 ms, flip angle = 70°, FOV = 256 mm, slice thickness = 2 mm, 63 slices, ARC factor = 2. There were 5 runs of fMRI (4 task, 1 CO_2_) acquired in total. A B_0_ field map was acquired for each fMRI run using a multi-echo fast spoiled gradient recalled echo sequence with TR = 500 ms, TE_1-4_ = 2.3/4.5/6.6/8.7 ms, flip angle = 30°, matched to the fMRI sequence. A sagittal 3D T_1_-weighted BRAVO structural image was acquired with TR/TE/TI = 6.7/2.9/650 ms, flip angle = 10°, matrix = 256 × 256, 1 mm^3^ isotropic voxels, 192 slices, ASSET factor = 2.

#### 2.2.3 Task description

The attention network task-revised (ANT-R) was the cognitive paradigm implemented in study 2 (Fan et al., 2005; Fan et al., 2009; Fan et al., 2012; Xuan et al., 2016). Attention is the cognitive domain that guides detection and prioritization of relevant features, and is comprised of 3 distinct networks: alerting, orienting and executive control (Petersen and Posner, 2012; Mackie et al., 2013). The ANT-R is the most recent version (at the time of testing) of a widely implemented behavioural paradigm that independently assesses the three attention networks and with fMRI, delineates the regions associated with each network (Fan et al., 2009; Xuan et al., 2016; Markett et al., 2022). The alerting network, responsible for anticipation and arousal, is associated with numerous widespread cortical regions including the anterior cingulate cortex, inferior occipital, precentral gyrus and parietal lobule, mid temporal and fusiform gyri, and superior frontal regions (Xuan et al., 2016). The orienting network is responsible for shifting focus to the prioritized stimuli, and has anatomical associations with the parietal lobule, frontal eye fields, the superior colliculi and thalamus. The executive control network identifies relevant information amongst competing, irrelevant stimuli, and is associated with the frontal regions including the anterior cingulate and lateral prefrontal cortex (Fan et al., 2009). The ANT-R was implemented because of these well-known anatomical correlates widely dispersed across the cerebral cortex.

The ANT-R is described in detail in (Fan et al., 2009) and (Xuan et al., 2016). The task is a cued flanker paradigm, where a central fixation cross and two rectangles to the left and right of the cross remain on the screen throughout the entirety of the task. For each trial, a row of 5 arrows is briefly presented (500 ms) in one of the two rectangles. The participants’ task is to indicate as rapidly as possible whether the centre (third) target arrow is pointing to the left or to the right. This is achieved with a button press of their dominant hand using either the index or middle finger. The centre arrow was either congruent with the other 4 arrows (pointing the same direction) or incongruent. Prior to the onset of the target arrows, a transient visual cue appeared in most trials (60 out of 72 trials, or 83.3%). The cue was a short (100 ms duration) brightening of the rectangle. There were 4 types of cue conditions: no cue, double cue (where both rectangles brightened), valid spatial cue (rectangle that was going to contain the target arrows brightened) and invalid spatial cue (rectangle that did not contain the target arrows brightened). The time delay between the cue and arrow targets was randomly assigned per trial as either 0, 400 or 800 ms. The baseline period between trials (the inter-stimulus interval) was pseudo-randomised and between 2,000 and 12,000 ms. There was a total of 72 trials per run, and with a total of 260 volumes collected per run, with each run lasting 7 min and 48 s. A total of 4 ANT-R runs were acquired per participant.

#### 2.2.4 CO_2_ challenge

An MR-compatible breathing circuit was used to achieve mild hypercapnia during the fMRI scan. The apparatus consisted of a non-rebreathing facemask with unidirectional valves for separate gas delivery and sampling of end-tidal gases (Tancredi et al., 2014; MacDonald et al., 2018). Outside the scanner, an automatic gas delivery system consisting of a Digital Flo-Box and Mass Flow Controllers (Sierra Instruments, Monterey, CA) and BIOPAC sampling equipment (BIOPAC Systems Inc., Goleta, CA) controlled gas delivery and sampling. BIOPAC modules CO2100C and O2100C continually sampled CO_2_ and O_2_ throughout the duration of the scan (sampling rate = 100 mL/min). The hypercapnia paradigm consisted of 2 min of delivered gas mixture with increased CO_2_ (gas mixture of 5% CO_2_ and 95% medical air) interspersed with 2 min of medical air, resulting in a total scan time of 6 min.

#### 2.2.5 fMRI preprocessing

The schematic summarizing the data analysis pipeline for experiment 2 is shown in Figure 1. All fMRIs underwent distortion correction using the B_0_ field maps and FieldMap Toolbox in SPM (Jezzard and Balaban, 1995; Jenkinson, 2003). Motion correction was performed in SPM12 (https://www.fil.ion.ucl.ac.uk/spm/). All within-subject fMRI runs (4 x ANT-R and 1 x CO_2_) were aligned using affine registration with Advanced Normalization Tools (ANTs) (Avants et al., 2011; Avants et al., 2014). Affine registration between each subject’s fMRIs and structural T_1_, and normalisation of the T_1_ image to the MNI template, was performed using Unified Segmentation in SPM12 (Ashburner and Friston, 2005). Deformation fields from the T_1_ image segmentation were applied to the fMRIs, which were normalised to the MNI template. Minimal spatial smoothing (4 mm full-width at half-maximum) was performed to improve signal to noise ratio. Smoothing was performed here but not in experiment 1 because of the differences in spatial resolution.

#### 2.2.6 fMRI first-level analyses

The general linear model for the ANT-R task included 16 task-related regressors, corresponding to cue condition (no cue, double cue, valid, invalid), the direction of the centre target arrow relative to the surrounding arrows (congruent, incongruent) and the target location (left rectangle, right rectangle). Only correct trials were modelled in these tasks regressors; incorrect trials were modelled separately as regressors of no interest. Six motion parameters were also included as regressors. Weighted linear contrasts calculated from the estimated beta images corresponded to the three attention networks. However, due to having the most robust and spatially extensive cortical activation, the alerting network was the contrast of interest for the present study. The alerting network was defined as the contrast of double cue relative to baseline, similar to prior work (Xuan et al., 2016).

First-level analysis of the hypercapnia paradigm modelled the BOLD signal change to increased CO_2_ using end tidal partial-pressure of CO_2_ from the CO_2_ module breathing traces. On a per-subject basis, the end tidal CO_2_ was extracted from the breathing trace and interpolated to match the temporal resolution of the fMRI time-series. The mean BOLD time-course was extracted from each subjects’ grey matter mask and cross-correlated with the end tidal CO_2_ trace. For the cross-correlations, the end tidal CO_2_ time-course was systematically shifted by 1 TR (1.8 s) until the maximum correlation between the mean grey matter BOLD time-course and end tidal CO_2_ was found. The end tidal CO_2_ with maximal BOLD correlation was entered into the general linear model as the regressor of interest. The six motion parameters were entered into the model as regressors of no interest.

#### 2.2.7 ROI analyses

The procedure for ROI definition was consistent with experiment 1. PSC maps were calculated from the alerting contrast and CO_2_ challenge using only these significant voxels. CVR maps were obtained by dividing the CO_2_ PSC maps by the maximum change in end tidal CO_2_ (mm Hg) during the CO_2_ challenge. ROI-averaged CVR values were input to the linear regression analyses, with ROI values greater than 3 mean absolute deviations removed from the analysis.The same procedure as study 1 was implemented for the linear regressions, where only ROIs with significant activation in at least 10 participants were considered.

#### 2.2.8 Group-level analyses

The aim and methods of the group-level analyses outlined in experiment 1 (section 2.1.7) were identical to experiment 2. The group analyses for experiment 2 were run with both voxel-wise CVR correction using the Biological Parametric Mapping toolbox, and at the ROI level using SPM only. All analyses used alerting contrast images.

## 3 Results

### 3.1 Experiment 1: Breath-hold CVR

#### 3.1.1 fMRI first-level analyses

The SCAP task produced robust bilateral activation across the insula and frontal and parietal lobes. The successful inhibition contrast from the stop-signal task significantly activated the superior temporal gyrus bilaterally, left insula, medial cingulate cortex, and right middle frontal gyrus. The task-switch paradigm resulted in significant activation in the left posterior-medial frontal gyrus, left inferior parietal lobule, bilateral calcarine gyrus, and left postcentral gyrus, insula, and lingual gyrus.

#### 3.1.2 ROI analyses

The anatomical ROIs ranged in mean volume from 1,016 (±284) mm^3^ for the left frontal pole to 30,102 (±2,639) mm^3^ for the left superior frontal gyrus. All 68 of the anatomical ROIs and their volumes are available in Supplementary Table S1.

**FIGURE 2:**
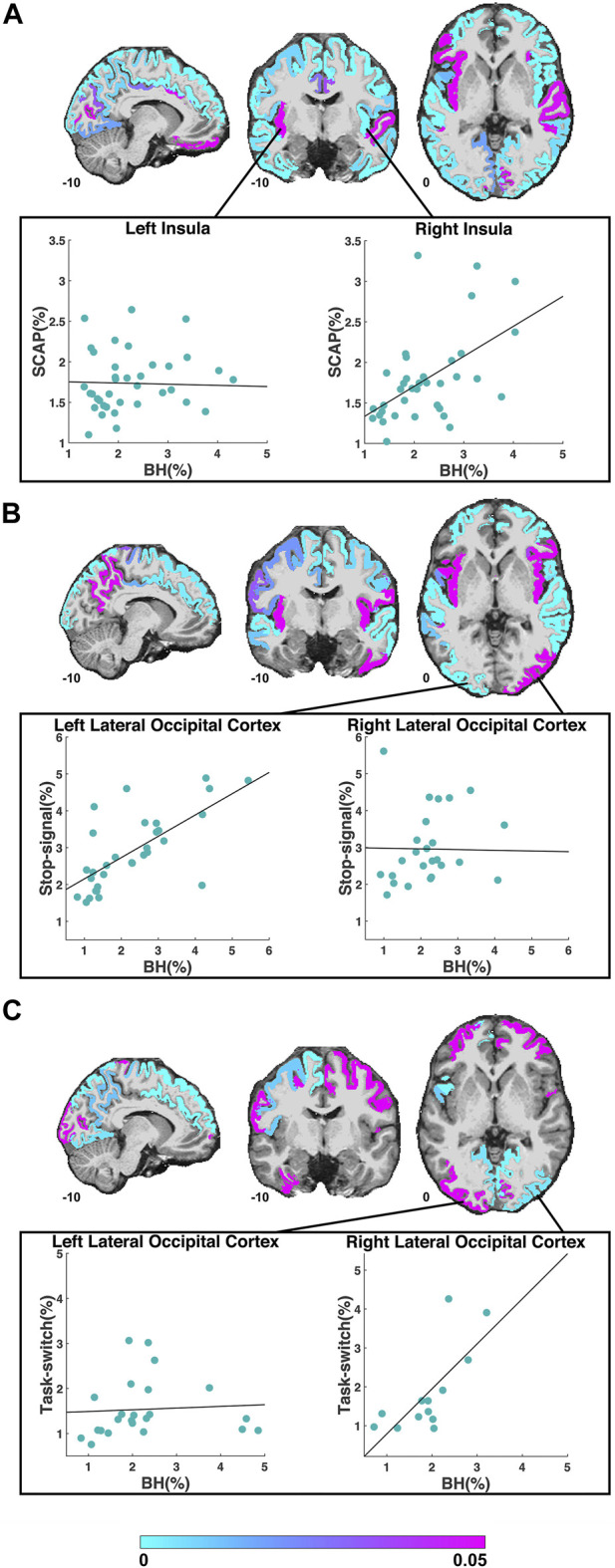
ROIs included in the regression analyses for the SCAP **(A)**, stop-signal **(B)** and task-switch **(C)** tasks are shown in cyan-magenta. ROIs where the task did not result in significant activation in at least 10 participants are not coloured. Images in coronal, sagittal and axial planes are shown in neurological orientation with MNI coordinates. The color bar indicates *p*-values from the regressions; regions in magenta did not show a significant linear relationship between breath-hold (BH) BOLD PSC and task PSC. The scatter plots in **(A)** demonstrate the PSC (breath-hold and SCAP) from the left and right insula to the SCAP task. The regression for the left insula (shown in magenta in the coronal plane) was non-significant, while the right insula (shown in cyan in the coronal plane) was significant. The scatter plots in **(B)** show an example of a significant (left lateral occipital cortex) and non-significant (right lateral occipital cortex) ROI for the stop-signal task. The scatter plots in **(C)** also show the left and right lateral occipital cortex, but to the task-switch paradigm. For this task, the regression for the left lateral occipital cortex was non-significant, while the right was significant. For all scatter plots, each data point represents one subject’s averaged PSC from that ROI.

The first cognitive task, the SCAP paradigm, had 51 ROIs with significant BOLD responses (to both SCAP and breath-hold) in 10 or more participants. The majority of these ROIs showed a significant linear relationship between SCAP activation and BOLD PSC to breath-hold, with 41 (80.1%) ROIs in total showing significant regression results (after FDR-correction for number of ROIs). ROIs and their corresponding original (prior to FDR-correction) *p*-values are displayed in Figure 2A; Table 1 outlines the top 10 ROIs with the strongest linear relationship between SCAP activation and breath-hold, as defined by *p*-values. The 10 ROIs that did not result in a significant regression are also outlined in Table 1. As shown in Table 1, these non-significant ROIs typically had a smaller sample size (i.e., fewer participants with significant activation in that ROI), as indicated by the ‘*n*’ column. There were exceptions however; one being the left insula. This ROI did not demonstrate a linear relationship between SCAP activation and breath-hold response but had similar activation and a larger *n* than the right insula, which did show a significant linear relationship (*p* = 0.0008, *R* ^2^ = 0.29, *n* = 36). The mean percent signal changes were similar between the two insula ROIs (right insula *M* = 1.79 and 2.24%, left insula *M* = 1.73 and 2.27% for SCAP and breath-hold respectively). Scatter plots for the left and right insula are shown in Figure 2A. All ROIs evaluated for the SCAP are given in the Supplementary Table S2.

**TABLE 1:** Regions-of-interest (ROI) with most significant (top 10) and non-significant (all) linear relationships between SCAP PSC and breath-hold PSC. *P* and *R* ^2^ values from the linear regression analyses shown.

ROI	*p*-value	*R* ^2^	*n*
*Top 10 significant ROIs*			
R precuneus	1.1 × 10^−8^	0.39	68
L superior temporal gyrus	5.3 × 10^−8^	0.57	38
R supramarginal gyrus	3.1 × 10^−7^	0.29	78
R inferior temporal gyrus	2.1 × 10^−6^	0.39	48
R inferior parietal cortex	3.2 × 10^−6^	0.23	85
R pars triangularis	7.9 × 10^−6^	0.48	33
L caudal middle frontal gyrus	1.3 × 10^−5^	0.30	54
L paracentral lobule	2.1 × 10^−5^	0.59	23
L supramarginal gyrus	4.7 × 10^−5^	0.17	90
L postcental gyrus	7.0 × 10^−5^	0.17	87
*Non-significant ROIs*			
L insula	0.84	0.001	39
L bank of the superior temporal sulcus	0.65	0.01	18
L pericalcarine cortex	0.32	0.05	23
R pericalcarine cortex	0.21	0.17	11
L caudal anterior cingulate	0.19	0.09	21
R bank of the superior temporal sulcus	0.11	0.20	14
L pars triangularis	0.09	0.13	23
L lateral orbitofrontal cortex	0.06	0.19	19
R lateral orbitofrontal cortex	0.05	0.12	32
R superior temporal gyrus	0.05	0.21	19

*R* ^2^ = coefficient of determination*, n* = number of participants.

R = right hemisphere, L = left hemisphere.

The second cognitive task, the stop-signal paradigm had 34 ROIs with adequate BOLD responses (>10 participants) for linear regression analyses. Out of these 34 ROIs, 25 (73.5%) showed a significant linear relationship between stop-signal BOLD activation and breath-hold response. These ROIs and their original *p*-values from the regression analyses are shown in Figure 2B. The scatter plots in this figure show an example significant and non-significant ROI. Table 2 outlines the top 10 ROIs with the strongest linear relationship, and the 9 ROIs that were non-significant. All evaluated ROIs are shown in the Supplementary Table S3.

**TABLE 2:** Regions-of-interest (ROI) with most significant (top 10) and non-significant (all) linear relationships between stop-signal PSC and breath-hold PSC. *P* and *R* ^2^ values from the linear regression analyses shown.

ROI	*p*-value	*R* ^2^	*n*
*Top 10 significant ROIs*			
R inferior parietal cortex	8.6 × 10^−9^	0.58	41
R superior temporal gyrus	1.8 × 10^−7^	0.38	60
R supramarginal gyrus	8.0 × 10^−7^	0.36	58
L supramarginal gyrus	1.6 × 10^−6^	0.42	45
L inferior parietal cortex	2.1 × 10^−6^	0.63	25
L superior parietal cortex	8.9 × 10^−6^	0.54	28
L lateral occipital cortex	1.6 × 10^−5^	0.48	31
R superior frontal gyrus	2.2 × 10^−5^	0.47	31
R middle temporal gyrus	3.4 × 10^−5^	0.36	41
R rostral middle frontal gyrus	6.0 × 10^−5^	0.38	37
*Non-significant ROIs*			
R lateral occipital cortex	0.91	0.0005	26
R inferior temporal gyrus	0.78	0.0006	14
R postcentral gyrus	0.58	0.02	15
L pars opercularis	0.48	0.07	10
R insula	0.15	0.12	18
L bank of the superior temporal sulcus	0.13	0.15	17
L insula	0.08	0.23	14
R pars triangularis	0.07	0.33	11
R lateral orbitofrontal cortex	0.04	0.36	12

*R* ^2^ = coefficient of determination*, n* = number of participants.

R = right hemisphere, L = left hemisphere.

The third cognitive task, the task-switch paradigm showed a total of 24 ROIs with adequate BOLD responses (in 10 or more participants) for linear regression analyses. Out of these, 11 ROIs (45.8%) were significant. These ROIs and their original *p*-values from the regression analyses are shown in Figure 2C; Table 3 demonstrates the top 10 ROIs with the strongest linear relationship, and all 13 ROIs that were non-significant. Similar to the previous two paradigms, the ROIs that did not show significant relationships between task-switch activation and breath-hold BOLD responses tended to demonstrate smaller *n* sizes. The exception was the left lateral occipital cortex, which was non-significant but had a larger *n* than the right lateral occipital cortex. The right, conversely, was highly significant. The mean PSC was similar between the two lateral occipital cortices (right *M* = 1.84 and 1.91%, and left *M* = 1.54 and 2.27% for task-switch and breath-hold respectively). The scatter plots comparing the left and right lateral occipital cortex are shown in Figure 2C. The linear regression results for all evaluated ROIs are given in the Supplementary Table S4.

**TABLE 3:** Regions-of-interest (ROI) with most significant (top 10) and non-significant (all) linear relationships between task-switch PSC and breath-hold PSC. *P* and *R* ^2^ values from the linear regression analyses shown.

ROI	*p*-value	*R* ^2^	*n*
*Top 10 significant ROIs*			
L superior frontal gyrus	1.8 × 10^−4^	0.62	17
L superior parietal cortex	0.0006	0.31	34
L pars opercularis	0.001	0.67	12
R lingual gyrus	0.002	0.31	28
R precuneus	0.002	0.45	18
L inferior parietal cortex	0.004	0.31	24
R lateral occipital cortex	0.005	0.53	13
L lingual gyrus	0.005	0.22	34
L precentral gyrus	0.01	0.23	28
L precuneus	0.01	0.34	18
*Non-significant ROIs*			
R pericalcarine	0.94	0.0006	11
R rostral middle frontal gyrus	0.85	0.003	16
L lateral occipital cortex	0.77	0.004	23
R superior frontal gyrus	0.44	0.08	10
L caudal middle frontal gyrus	0.39	0.06	15
L postcentral gyrus	0.30	0.06	19
R inferior parietal cortex	0.21	0.11	17
L fusiform	0.14	0.13	18
R precentral gyrus	0.09	0.22	14
L rostral middle frontal gyrus	0.08	0.13	24
R superior parietal cortex	0.06	0.17	22
L pericalcarine	0.05	0.21	19
R supramarginal gyrus	0.04	0.37	12

*R* ^2^ = coefficient of determination*, n* = number of participants.

R = right hemisphere, L = left hemisphere.

The regions that showed a significant linear relationship between task and breath-hold BOLD responses for all 3 cognitive tasks were the left inferior parietal cortex, precentral gyrus, superior frontal gyrus, and supramarginal gyrus; and the right precuneus. These ROIs are displayed in the Supplementary Figure S1. The ROIs with adequate responses (significant activation to at least one task and breath-hold in >10 participants) but failed to show significant regressions in any of the 3 tasks were the left bank of the superior temporal sulcus, left caudal anterior cingulate cortex, left insula, and left pars triangularis; the pericalcarine cortex bilaterally, and the lateral orbitofrontal cortex bilaterally.

#### 3.1.3 Group-level analyses

**FIGURE 3:**
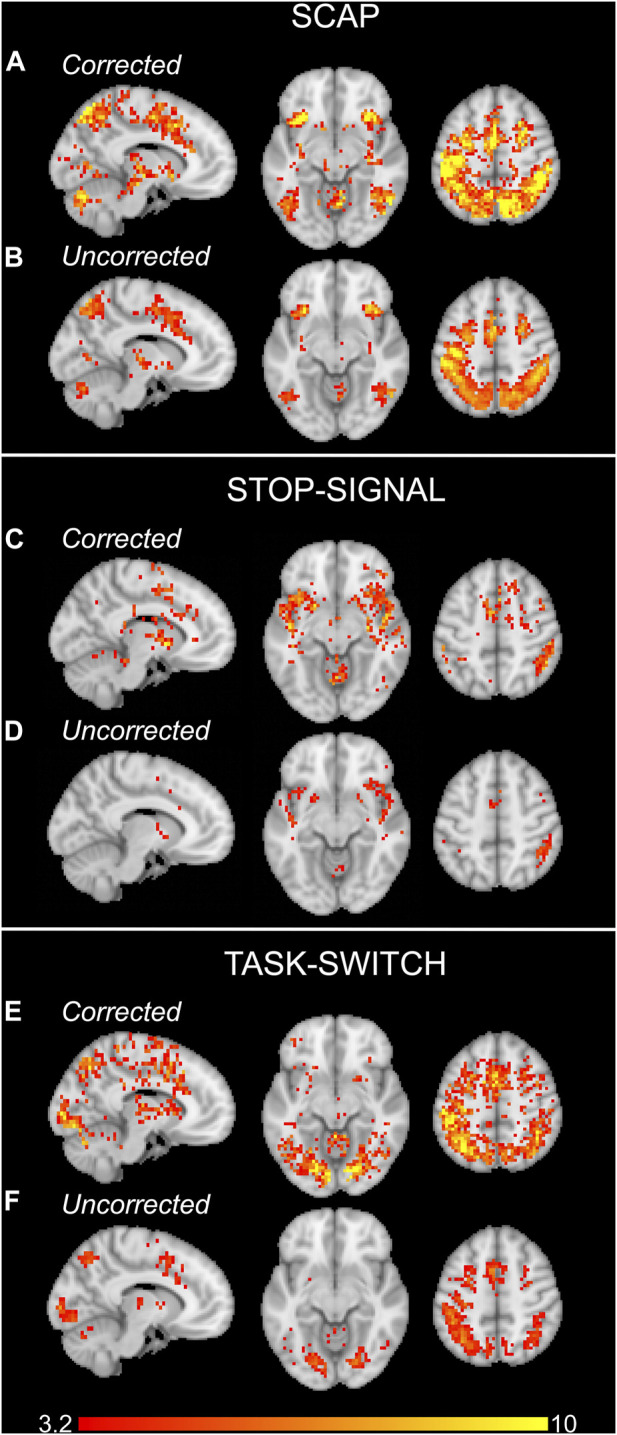
Group activation maps for the SCAP **(A, B)**, stop-signal **(C, D)** and task-switch **(E, F)** paradigms for experiment 1. The upper rows of each task **(A, C, E)** show the activation maps which were corrected for CVR by including the breath-hold contrast images as covariates. The lower row for each task **(B, D, F)** are the standard, uncorrected for CVR, group activation maps. All images cluster-corrected for multiple comparisons (*p* < 0.05). The colour bar indicates t-values.

Activation maps in Figure 3 show the group activation to the SCAP, stop-signal and task-switch paradigms both with and without CVR correction using the voxel-wise image covariate approach. Figures 3A, C, E show maps that have been corrected for CVR by including breath-hold as an image covariate in the Biological Parametric Mapping toolbox, and Figures 3B, D, F demonstrate the standard analyses without correction (i.e., no image covariate). For all 3 tasks, the corrected group-level analyses showed a greater number of significant voxels than their uncorrected counterparts. The corrected activation maps also had higher peak voxel *t*-values for SCAP, stop-signal and task-switch (*t* = 18.65, 13.69 and 15.00 respectively) compared to the uncorrected maps (*t* = 12.63, 7.84, 8.50). The ROI approach was also applied to correct for CVR, however there were no differences between CVR corrected and uncorrected activation maps for this method.

### 3.2 Gas inhalation CVR

#### 3.2.1 CO_2_ challenge

At baseline when participants were exposed to medical air only, the mean end tidal CO_2_ was 35.38 (±4.1) mmHg (elevation of testing location was 1,100 m). During the 2-min hypercapnia period when participants were exposed to 5% CO_2_, the mean maximum end tidal CO_2_ was 45.19 (±3.59) mmHg. The mean change in end tidal CO_2_ between baseline and hypercapnia was 9.81 (±3.06) mmHg.

#### 3.2.2 fMRI analyses

**FIGURE 4:**
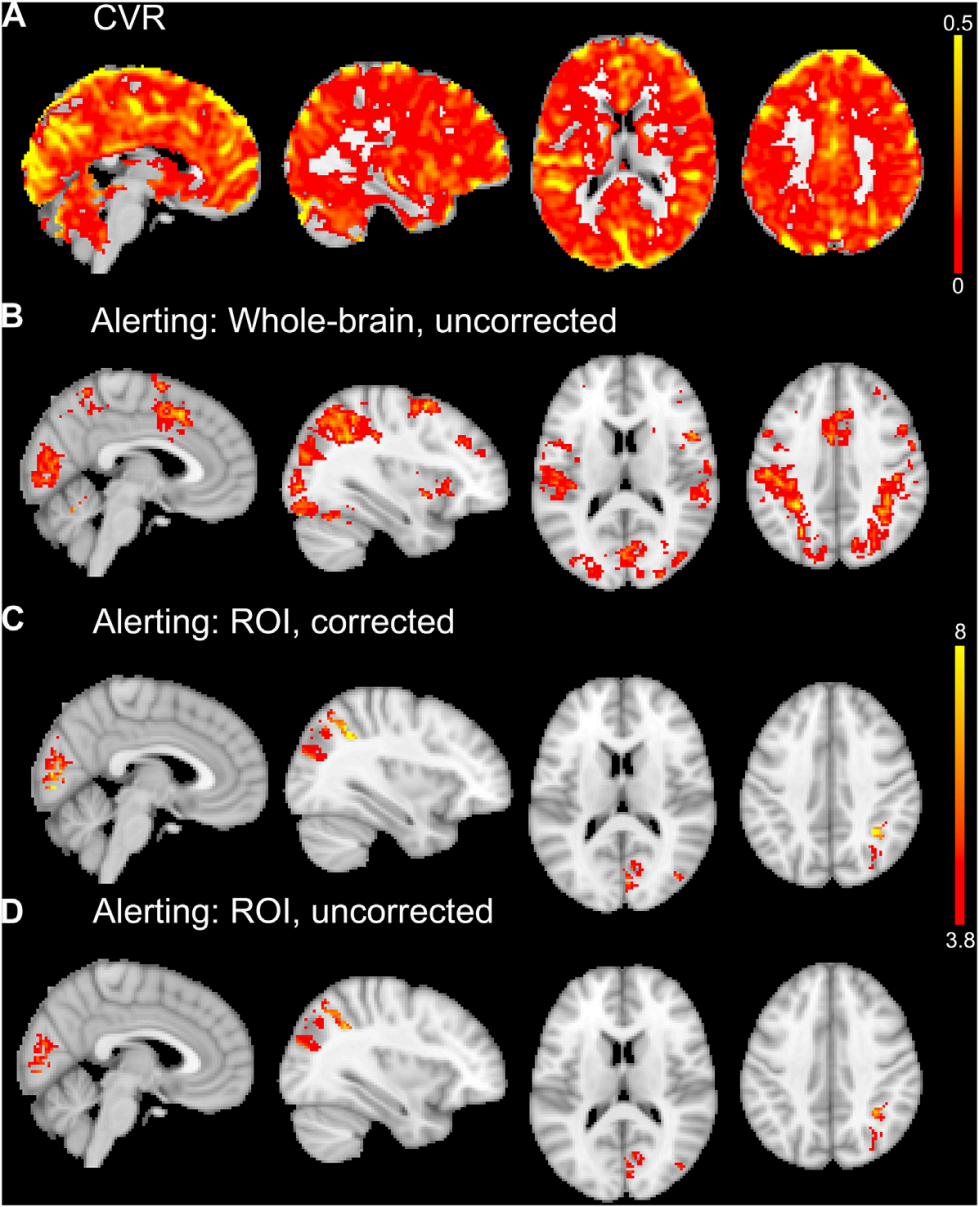
An example CVR map of a single subject is shown in **(A)**. Whole-brain average group maps for the alerting contrast of the ANT-R shown in **(B)**. The ROI analyses of the alerting contrast shown in **(C)** and **(D)** included explicit masks comprised of the five significant ROIs from the linear regression analyses for experiment 2. For the vascular corrected ROI analysis in **(C)**, the mean CVR values for each ROI were entered into the model as covariates. The vascular uncorrected ROI analysis shown in **(D)** included the explicit masks only with no covariates. The group maps in **(B, C, D)** were thresholded at *p* < 0.001 uncorrected for multiple comparisons. The upper colour bar indicates %BOLD/mmHg for the CVR map in **(A)**. The lower colour bar indicates t-values for **(B, C, D)**.

The alerting contrast produced robust activation across the cerebral cortex with peak activation in the left inferior parietal lobule, right inferior frontal gyrus, left fusiform gyrus, left posterior medial-frontal gyrus, right inferior occipital gyrus, right superior parietal lobule, and right middle frontal gyrus. The CO_2_ challenge produced increased BOLD responses across the cerebral cortex, particularly from the large sinuses. An example CVR map is shown in Figure 4A, and the group activation map to the alerting contrast is shown in Figure 4B.

#### 3.2.3 ROI analyses

The mean volumes of the anatomical ROIs ranged from 820 (±255) mm^3^ for the right transverse temporal cortex to 25,196 (±4,472) mm^3^ for the left superior frontal gyrus. All 68 of the anatomical ROIs and their mean volumes are displayed in Supplementary Table S1.

**FIGURE 5:**
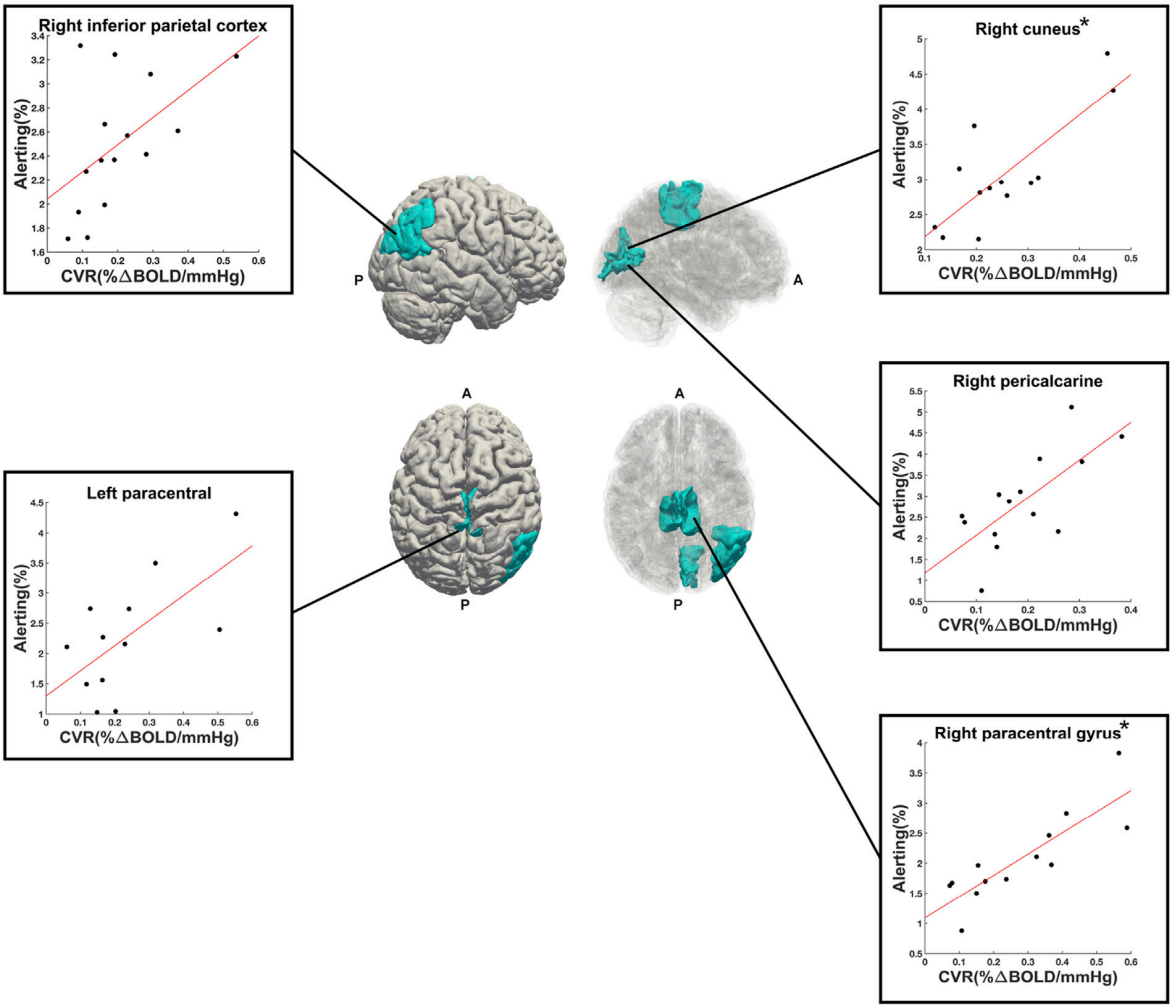
Right (upper) and superior (lower) views of the significant ROIs (in cyan) from the alerting contrast of the ANT-R task. Reduced opacity images on the right highlight medial aspects. Scatter plots show the linear relationship between alerting PSC (*y*-axis) and CVR (*x*-axis) for each ROI. The (*) next to right cuneus and right paracentral gyrus indicate that these two ROIs were significant following FDR-correction for multiple comparisons. A = anterior, P = posterior.

A total of 50 ROIs had significant activation in 10 or more participants and were entered into linear regression analyses. Out of these 50 ROIs, 5 showed an uncorrected (*p* < 0.05, before FDR-correction for multiple comparisons) significant linear relationship between alerting activation and CVR: right paracentral gyrus (*p* = 0.0003, *R* ^2^ = 0.71, *n* = 13), right cuneus (*p* = 0.001, *R* ^2^ = 0.64, *n* = 13), right pericalcarine (*p* = 0.004, *R* ^2^ = 0.51, *n* = 14), left paracentral lobule (*p* = 0.02, *R* ^2^ = 0.42, *n* = 12), and right inferior parietal cortex (*p* = 0.04, *R* ^2^ = 0.28, *n* = 15). The first two (right paracentral gyrus and cuneus) were the only ROIs to survive FDR correction for multiple comparisons. These five ROIs are shown in Figure 5, along with their respective scatter plots. The linear regression results from all evaluated ROIs can be found in the Supplementary Table S5.

**FIGURE 6:**
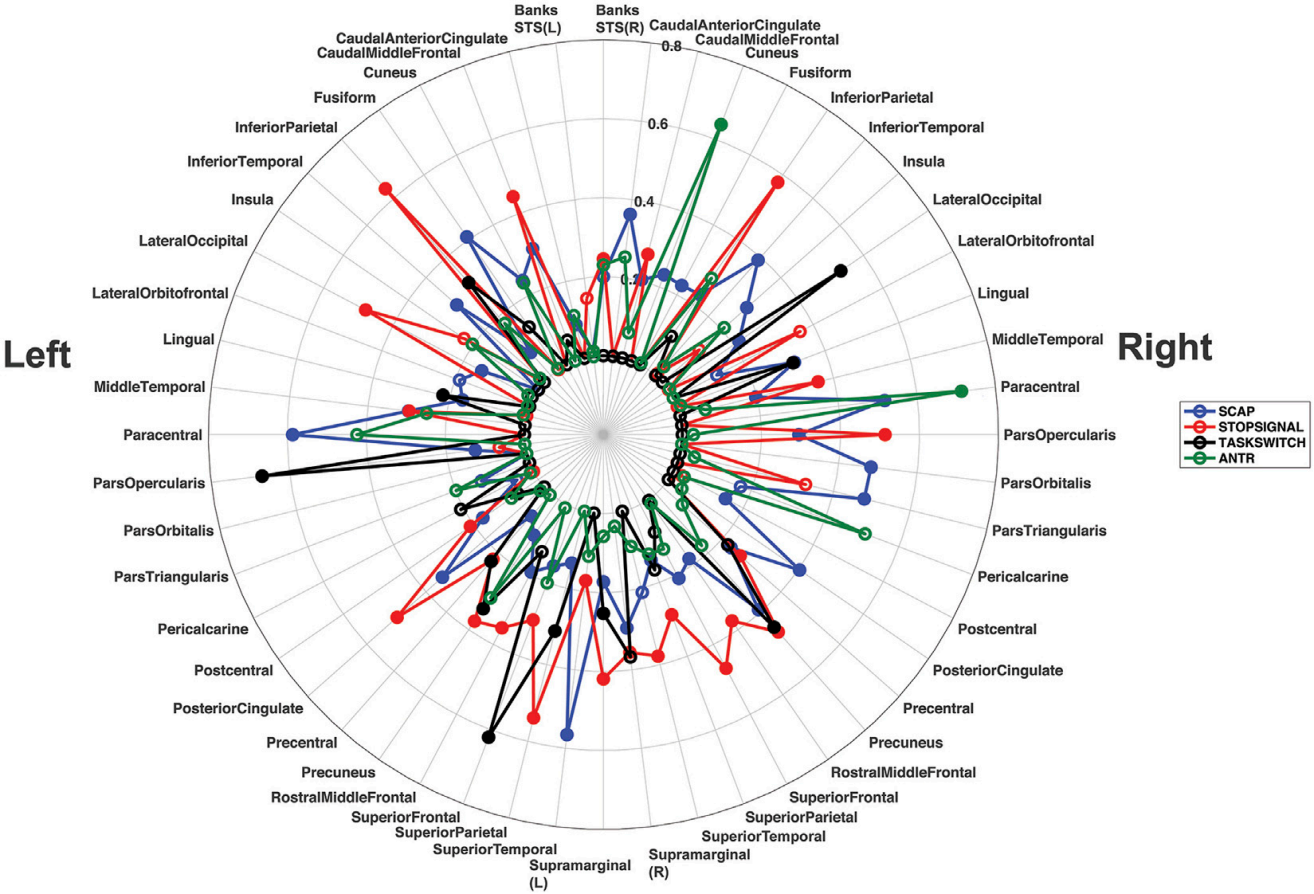
Polar plot displaying the *R* ^2^ values for all ROIs from all linear regression analyses, across both experiments. Each ROI shows its corresponding *R* ^2^ value for the left and right hemisphere separately. The *R* ^2^ values for the SCAP are shown in blue, stop-signal in red, task-switch in black and ANT-R in green. The open-coloured markers (‘o’) indicate that the ROI was not significant after FDR correction for multiple corrections, while the closed markers were significant. The concentric circles indicate the *R* ^2^ value, with values increasing with radius to a maximum of *R* ^2^ = 0.80.

*R* ^2^ values from all ROIs, from both studies (breath-hold and gas-induced hypercapnia) are displayed in a polar plot in Figure 6. This plot indicates that when activated by a task, most ROIs showed at least a moderate linear relationship between task-induced signal magnitude and CVR. Low correspondence between ROI activation and CVR (indicated by small *R* ^2^ on Figure 6) typically reflects lack of activation by the task.

#### 3.2.4 Group-level analyses

The first approach to CVR correction using voxel-wise general linear models and the Biological Parametric Mapping toolbox was not optimal for experiment 2, as it resulted in less activation than the uncorrected (i.e., standard one-sample *t*-test) group analysis. The second ROI approach to CVR correction implemented only the five significant ROIs from the linear regression analyses (right paracentral gyrus, right cuneus, right pericalcarine, left paracentral lobule, right inferior parietal cortex). This ROI approach resulted in small increases in the number of significant voxels (at *p* < 0.001 uncorrected for multiple comparisons) for the vascular corrected compared to the uncorrected analyses. The largest differences were observed in the right inferior parietal cortex (+27 voxels for vascular corrected vs. uncorrected) and right cuneus (+24 voxels for vascular corrected vs. uncorrected). There were increases in the peak *t*-values for most of the vascular corrected ROIs compared to the uncorrected ROIs (right cuneus: *t* = 8.94 and 6.36; right pericalcarine: *t* = 8.78 and 7.03; left paracentral lobule: *t* = 6.26 and 6.49; right inferior parietal cortex: *t* = 9.33 and 7.25 for vascular corrected and uncorrected respectively). The right paracentral gyrus is unreported as there were no suprathreshold voxels for both vascular corrected and uncorrected analyses. Activation maps from the vascular corrected and uncorrected ROI analyses are shown in Figures 4C, D respectively.

## 4 Discussion

This research aimed to evaluate whether basal vascular physiology, assessed using CVR, can predict task-induced BOLD responses across multiple cognitive tasks activating different cortical regions. The hypothesis that linear relationships between CVR and task-based BOLD responses would be observed across all brain regions evaluated was largely supported. Evidence for this can be found in the polar plot in Figure 6, showing that the majority of regions investigated showed at least a moderate (*R* ^2^ > 0.20) relationship between BOLD activation magnitude and CVR to at least one task. These findings highlight that the relationship between neural activity-mediated BOLD signal and vascular physiology is mostly preserved across the cerebral cortex, to different cognitive tasks and with different CVR approaches.

For experiment 1, task activation was predicted by breath-hold BOLD responses for the majority of evaluated ROIs, although some exceptions were found. Most of these exceptions could be explained by smaller sample size, as fewer participants demonstrated significant task and/or breath-hold BOLD responses compared to ROIs that were significant. This relationship between variance explained by breath-hold responses and sample size can be appreciated from the information presented in Tables 1–3 where both *R* ^2^ and ‘*n*’ are reported for significant and non-significant ROIs. There were two exceptions found, however. For the SCAP task, a paradigm of spatial memory, the insula showed strong BOLD responses bilaterally, however, only activation from the right insula showed a linear relationship with CVR. Likewise, for the task-switch paradigm, the lateral occipital cortex showed strong bilateral BOLD responses but only the right hemisphere showed a significant linear relationship with CVR. These two non-significant ROIs (left insula and lateral occipital cortex) cannot be fully explained by small sample size, as they had more participants with significant activation than their contralateral counterpart. Differences in mean PSC between these ROIs is another potential explanation but does not appear relevant here as these were also highly consistent between hemispheres. Moreover, the left insula failed to show a significant linear relationship with CVR across all paradigms; this includes the SCAP, the stop-signal task with a sample size of 14 participants, and the ANT-R with 12 participants. Similarly, the right insula was activated by the stop-signal (*n* = 18) and ANT-R (*n* = 13) tasks but was non-significant in the ROI analyses. Bilaterally the insula was not activated by the task-switch paradigm and was not included in the linear regression analyses for this task. One possible explanation is that left insula activation to the SCAP task and its relationship with breath-hold BOLD responses varied across participants of different ages. Further analyses were therefore run, separating the youngest age group (participants aged in their 20’s at time from testing) from the oldest age group (people aged 40–50 years at time of testing). The scatter plot shown in the Supplementary Figure S2, shows a difference between these two groups which may explain the non-significant finding. Supplementary Figure S2 shows a negative (albeit non-significant) relationship between task-based activation and breath-hold BOLD responses for the older group, while the younger group showed a significant positive relationship. The present study lacks the sample size to fully investigate this age effect observed in the left insula, however it is hoped that these preliminary data provide impetus for future research.

Unlike the insula, the lateral occipital cortex showed a more task-dependent relationship with CVR. For this region, the left hemisphere regression (with breath-hold as the predictor variable) was significant in the stop-signal task (*n* = 31) but the right was not (*n* = 26). This was the opposite for the task-switch paradigm, where the left was non-significant (*n* = 23) and the right significant (*n* = 13). For the SCAP, both the right (*n* = 44) and the left (*n* = 53) lateral occipital cortices were significant. Both left and right lateral occipital cortices were non-significant in the ANT-R (*n* = 15 for both). Unlike the insula, this appears to be a region that has strong correspondence with vascular physiology. However, task-related variability in how each task recruited this region, and the strength with which each participant individually recruited this region, was observed.

The regions that showed significant linear regressions across all three cognitive paradigms in experiment 1 were mainly left-lateralized and showed parietal lobe dominance. Out of the 5 ROIs that were consistently significant across the three tasks, only one was outside the parietal region: the left superior frontal gyrus. This left-lateralized consistency may reflect strong BOLD responses within this region due to the common task requirement, a motor response (button press) with their dominant hand. However, the bilateral parietal lobes were dominant in experiment 2. For the five ROIs significant at *p* < 0.05 (uncorrected for multiple comparisons) in this gas-inhalation study, three were in the parietal lobe (two right hemisphere). There was a button-press response required by the dominant hand for the ANT-R task, and all participants in this study were right-handed. Motor responses might explain some of the left parietal dominance (for example, the left paracentral ROI in the ANT-R task), however further consideration is required as the inferior parietal cortex (IPC) is spatially discrete from the motor cortex. The IPC was the only region to be significantly related to CVR across all four tasks, in the two separate experiments, with the left IPC in study 1 and the right IPC in experiment 2. Regions such as the parietal cortex where activation was strongly and consistently predicted by CVR might be particularly prone to confounding task-related BOLD signal changes due to CVR, such as those observed in aging.

Brain aging leads to cerebrovascular changes, with vascular stiffening and endothelial dysfunction compromising a vessel’s ability to respond to CO_2_ (Abdelkarim et al., 2019). CVR decline with advancing age has been reported (Bhogal et al., 2016; McKetton et al., 2018; Peng et al., 2018). Changes in neural activity-mediated BOLD signal also change with age (West et al., 2019), and disentangling the neural and non-neural contributions is vital for the appropriate interpretation of BOLD fMRI (Tsvetanov et al., 2021b). Addressing age-related vascular physiology differences is an area where vascular correction such as hypercapnic normalization should be considered essential. In these applications however, it is important to consider partial voluming and the increased contribution of cerebrospinal fluid. This might be a particularly important consideration in studies of aging where cortical atrophy occurs, increasing the likelihood of voxel cerebrospinal fluid contributions. In these instances, factors to prevent partial voluming such as increased spatial resolution should be considered. However, there are other applications outside of aging that would benefit from correction of baseline physiology. For example, acute sleep deprivation in healthy subjects has shown to increase regional cerebral blood flow (Elvsåshagen et al., 2019). Evidence for CVR changes due to acute sleep deprivation is lacking, however chronic sleep deprivation in subjects with obstructive sleep apnea has shown to be associated with increases in CVR (Ryan et al., 2018). Differences in habitual sleep patterns are a likely source of BOLD inter-subject variability, and the variability due to vascular changes might be addressed with vascular correction. Similarly, caffeine is a known vasoconstrictor that affects fMRI activation (Mulderink et al., 2002; Laurienti et al., 2003; Griffeth et al., 2011; Specht et al., 2019), however research addressing whether vascular correction can address vascular changes is lacking and highly warranted (Williams et al., 2021a). It is possible that medication can also affect BOLD signal, although this is medication-dependent and only certain medications have, to date, been evaluated in various patient groups (Goozee et al., 2016; Delfin et al., 2020; Williams et al., 2021b). Nonetheless, vascular correction in studies comparing medication-taking versus medically naive groups would improve interpretation of BOLD signal changes by separating variability due to vascular and non-vascular factors. Vascular correction might also improve the accuracy of BOLD fMRI magnitudes to scale with neural activity, such as experimental designs with increasing cognitive load or sensory load (for example, visual studies with partial to full visual field stimulation). This is because the vascular corrected activation map might have reduced dependence on larger blood vessels, thereby improving the accuracy of the map to reflect neural activity. Further research on this is required.

An important area of research that might benefit from vascular correction includes comparisons of patient and healthy control groups where cerebrovascular changes due to disease processes in the patient group produce confounding results. This has been highlighted in patients with Moyamoya disease where significant arterial stenosis changes the distribution of cerebral blood flow during activation and hypercapnia (Mazerolle et al., 2018; Hauser et al., 2019). One consideration is whether patients with CVR impairment will demonstrate a linear response to CO_2_. There is evidence supporting a sigmoidal rather than linear relationship between CVR response and end-tidal partial pressure of CO_2_. In a study of healthy participants, Bhogal et al. (2014) showed that the linearity between BOLD percent signal change and end-tidal CO_2_ did not hold at high CO_2_ stimulation (Bhogal et al., 2014). Patients with impaired vascular reactivity due to vessel pre-dilation may demonstrate a shift in the sigmoidal response such that BOLD responses are diminished at weaker levels of CO_2_ stimulation. With hypercapnic normalization, it’s assumed that a pre-dilated vessel would show a corresponding diminished BOLD activation to a task. It has been shown that task-based BOLD activation is reduced when baseline vasodilatory potential is reduced due to increased baseline end-tidal CO_2_ (van Niftrik et al., 2018), suggesting that hypercapnic normalization would be ideally implemented in participants with impaired vascular reactivity.

When discussing fMRI vascular correction using hypercapnia-based approaches such as CVR, it is critical to address the assumption of iso-metabolism. When CVR studies are performed, it is assumed that metabolism remains consistent across hypercapnic and normocapnic epochs, however recent research findings have challenged this. Deckers et al. (2022) reported a mean decrease of 13.4% in cerebral metabolic rate of oxygen during inhalation of 5% CO_2_ in air (Deckers et al., 2022). This is a concern for calibrated fMRI studies that aim to calculate cerebral metabolic rate of oxygen (Blockley et al., 2015), and it should also be a consideration for CVR studies using BOLD. The BOLD amplitude will be overestimated in the context of reduced deoxyhemoglobin concentrations, thereby overestimating the calculated CVR. Alternative approaches for acquiring CVR maps for vascular correction might include the use of arterial spin labelling rather than BOLD, and altering the gas mixtures to induce a slight hypoxic component (Peng et al., 2017). There is emerging research suggesting that CVR can be calculated from gas-free methods such as resting-state BOLD, which is also a possibility for future research using vascular correction (Wise et al., 2004; Kannurpatti and Biswal, 2008; Kannurpatti et al., 2012; Tsvetanov et al., 2015; Golestani et al., 2016a; Golestani et al., 2016b; Kazan et al., 2016; Wang et al., 2016; Jahanian et al., 2017; Liu et al., 2017; Pinto et al., 2020; Tsvetanov et al., 2021a; Stickland et al., 2021; Addeh et al., 2023). Other alternatives to the breath-hold and fixed concentration CO_2_ hypercapnic stimuli that were utilised in the current study are targeted gas systems, where an end-tidal CO_2_ value above baseline is targeted. This approach may provide further benefit to hypercapnic normalization as it reduces the variability associated with arterial CO_2_ concentrations, which may then reduce inter-subject variation in CVR maps (Slessarev et al., 2007).

The group analyses in experiment 1 showed that vascular correction at the group level, performed by including the individual breath-hold contrast images as voxel-wise covariates, increased sensitivity to BOLD signal change as indicated by both the number of significant voxels and the peak *t*-values. The ROI approach to vascular correction had no effect on the activation maps from experiment 1, but in experiment 2 this approach resulted in minor improvements for the CVR corrected maps through increases in *t*-values. Linear regression analyses from experiment 2 revealed only five ROIs that predicted BOLD activation from CVR, which might explain why the whole-brain voxel-wise covariate approach that benefitted experiment 1 did not benefit group analyses in experiment 2. This raises the question of whether vascular correction applied in a voxel-wise manner only benefits larger sample sizes, as experiment 1 had a much larger sample size than experiment 2. Sample size is one contributing factor, but others should also be considered when determining whether vascular correction is worthwhile. Groups with substantial inter-subject variability due to vascular physiology would benefit more from vascular correction than highly homogenous groups, even when sample sizes are small. In first-level modelling, precision in the estimate of the effect of interest is another pertinent factor, and this will depend on within-subject variability. This, in turn, is highly dependent on the task implemented and the number of runs, conditions, and if a cognitive task-the number of correct responses and subject variability due to attention and habituation (Chen et al., 2012). The hardware (e.g., scanner field strength and head coil) and software (e.g., use of simultaneous multi-slice, parallel imaging) might also vary the effect estimate when comparing across datasets and sites.

The small sample size for experiment 2 (*n* = 15) is a limitation of the present study, and the relatively smaller number of significant ROIs (5 out of 50 at *P* < 0.05, and 2 out of 50 after FDR-correction) compared to experiment 1 may have reflected this. Another limitation to the present study is that the voxel selection process for the linear regression analyses meant that only robustly activated brain regions were included. This could inflate *R* ^2^ values due to decreased inter-subject variability, a result of choosing only voxels above a set threshold. The alternative would be to include all voxels regardless of whether they were activated by a task; however, this alternative was considered less optimal as investigating whether task magnitudes could be predicted by CVR require a hemodynamic response.

In summary, the present study showed that CVR is mostly predictive of cognitive task-based fMRI activation across the cerebral cortex. The parietal regions were consistently related to CVR across all tasks and CVR approaches, suggesting that BOLD activations in these areas scale with baseline vascular physiology and might be prone to BOLD signal variability due to vascular changes. Most regions that were strongly activated by a task also showed a significant relationship with CVR; although, the left insula was one activated region that did not show this relationship. For the left insula, non-vascular factors such as neural variability might better explain inter-subject variability. The group analyses showed that including vascular covariates increased statistical significance of activation maps. Overall, these results provide support for the close relationship between CVR and BOLD response magnitude to complex fMRI paradigms. This suggests that vascular correction techniques such as hypercapnic normalization should be considered for all fMRI paradigms to disentangle vascular and non-vascular variability in BOLD signal magnitude.

## Data Availability

The raw data supporting the conclusion of this article will be made available by the authors, without undue reservation.
