# Supplementary material for: Correspondence between BOLD fMRI task response and cerebrovascular reactivity across the cerebral cortex
Source: Front Physiol. 2023 May 9;14:1167148. doi: 10.3389/fphys.2023.1167148 (PMC10203231; doi:10.3389/fphys.2023.1167148)
Supplement: Supplementary file 1 [file DataSheet1.docx]

# Supplementary Materials

**Table S1.** Mean volumes (with standard deviations in parentheses) in mm^3^ of the cortical parcellations used as anatomical ROIs across all subjects for experiments 1 (breath-hold, *n* = 114) and 2 (gas-inhalation, *n* = 15). ROIs are shown in alphabetical order, with left and right hemisphere means volumes shown separately.

| **Anatomical ROI** | **Experiment 1: Left** | **Experiment 1: Right** | **Experiment 2: Left** | **Experiment 2: Right** |
| --- | --- | --- | --- | --- |
| Bank of the superior temporal sulcus | 3369 (540) | 3173 (460) | 2648 (609) | 2480 (563) |
| Caudal anterior cingulate | 2441 (524) | 2829 (581) | 1878 (732) | 2242 (786) |
| Caudal middle frontal gyrus | 8601 (1303) | 7795 (1242) | 7375 (1227) | 6774 (1398) |
| Cuneus | 3595 (483) | 3876 (498) | 2859 (677) | 2935 (674) |
| Entorhinal cortex | 2489 (432) | 2311 (467) | 2200 (525) | 1979 (506) |
| Frontal pole | 1016 (184) | 1337 (215) | 883 (333) | 1026 (346) |
| Fusiform | 12579 (1402) | 12649 (1299) | 9452 (2259) | 8518 (1971) |
| Inferior parietal cortex | 16248 (1847) | 19673 (1981) | 12193 (2048) | 14614 (2993) |
| Inferior temporal gyrus | 14063 (1821) | 14107 (1694) | 12640 (2397) | 11895 (1982) |
| Insula | 8859 (836) | 9034 (757) | 7160 (1395) | 7456 (2791) |
| Isthmus cingulate cortex | 3443 (468) | 3136 (424) | 2268 (886) | 1657 (1000) |
| Lateral occipital cortex | 14844 (1586) | 14859 (1619) | 12811 (3439) | 13525 (6409) |
| Lateral orbitofrontal cortex | 9561 (806) | 9108 (855) | 8406 (1420) | 8249 (1557) |
| Lingual gyrus | 8377 (996) | 8661 (911) | 6125 (2229) | 5547 (1846) |
| Medial orbitofrontal cortex | 6293 (711) | 6304 (617) | 5700 (1128) | 5809 (1088) |
| Middle temporal gyrus | 14421 (1570) | 16104 (1642) | 11630 (2123) | 12984 (2589) |
| Paracentral lobule | 4457 (1570) | 5105 (669) | 3381 (901) | 3646 (783) |
| Parahippocampal gyrus | 2888 (413) | 2813 (400) | 2818 (2146) | 1697 (946) |
| Pars opercularis | 6279 (871) | 5200 (819) | 4571 (910) | 4227 (898) |
| Pars orbitalis | 2858 (313) | 3415 (437) | 2633 (483) | 3008 (675) |
| Pars triangularis | 4685 (671) | 5488 (796) | 3966 (940) | 4477 (906) |
| Pericalcarine | 2688 (372) | 3059 (363) | 2057 (490) | 2251 (970) |
| Postcentral gyrus | 12573 (1346) | 11479 (1451) | 10274 (1980) | 9371 (1350) |
| Posterior cingulate cortex | 4212 (543) | 4301 (664) | 2943 (849) | 2847 (914) |
| Precentral gyrus | 17453 (1571) | 17115 (1474) | 14315 (2752) | 13327 (2369) |
| Precuneus | 12358 (1146) | 12856 (1216) | 9120 (2353) | 8843 (2322) |
| Rostral anterior cingulate | 3393 (523) | 2678 (468) | 2371 (877) | 2101 (849) |
| Rostral middle frontal gyrus | 20479 (2046) | 20549 (2003) | 16466 (3513) | 16542 (2755) |
| Superior frontal gyrus | 30102 (2639) | 28551 (2526) | 25196 (4472) | 23971 (4306) |
| Superior parietal cortex | 17072 (1929) | 17263 (1736) | 13875 (2753) | 13569 (2582) |
| Superior temporal gyrus | 16253 (1572) | 15365 (1398) | 13814 (2321) | 12543 (2902) |
| Supramarginal gyrus | 14418 (1635) | 13478 (1540) | 11870 (2631) | 10089 (1992) |
| Temporal pole | 3063 (427) | 2879 (466) | 2291 (714) | 2335 (688) |
| Transverse temporal cortex | 1536 (256) | 1207 (203) | 1177 (382) | 820 (255) |

**Table S2.** All regions-of-interest (ROI) evaluated in linear regression analyses for SCAP PSC and BH PSC. *P* and R^2^ values from the linear regression analyses shown.

| **ROI** | ***P*-value** | **R^2^** | ***n*** |
| --- | --- | --- | --- |
| R precuneus | 1.1 x 10^-8^ | 0.39 | 68 |
| L superior temporal gyrus | 5.3 x 10^-8^ | 0.57 | 38 |
| R supramarginal gyrus | 3.1 x 10^-7^ | 0.29 | 78 |
| R inferior temporal gyrus | 2.1 x 10^-6^ | 0.39 | 48 |
| R inferior parietal cortex | 3.2 x 10^-6^ | 0.23 | 85 |
| R pars triangularis | 7.9 x 10^-6^ | 0.48 | 33 |
| L caudal middle frontal gyrus | 1.3 x 10^-5^ | 0.30 | 54 |
| L paracentral lobule | 2.1 x 10^-5^ | 0.59 | 23 |
| L supramarginal gyrus | 4.7 x 10^-5^ | 0.17 | 90 |
| L postcental gyrus | 7.0 x 10^-5^ | 0.17 | 87 |
| R precentral gyrus | 1.0 x 10^-4^ | 0.23 | 60 |
| R superior frontal gyrus | 1.3 x 10^-4^ | 0.21 | 64 |
| L fusiform | 1.9 x 10^-4^ | 0.41 | 29 |
| R superior parietal cortex | 2.2 x 10^-4^ | 0.14 | 93 |
| L superior parietal cortex | 2.3 x 10^-4^ | 0.13 | 96 |
| R pars opercularis | 4.3 x 10^-4^ | 0.29 | 38 |
| R caudal anterior cingulate | 4.4 x 10^-4^ | 0.36 | 30 |
| R rostral middle frontal gyrus | 6.2 x 10^-4^ | 0.18 | 61 |
| L superior frontal gyrus | 7.2 x 10^-4^ | 0.16 | 70 |
| R insula | 8.0 x 10^-4^ | 0.28 | 36 |
| R caudal middle frontal gyrus | 8.7 x 10^-4^ | 0.20 | 51 |
| L rostral middle frontal gyrus | 8.9 x 10^-4^ | 0.19 | 54 |
| L inferior temporal gyrus | 0.0013 | 0.30 | 32 |
| R lateral occipital cortex | 0.0015 | 0.22 | 44 |
| R paracentral gyrus | 0.0038 | 0.52 | 14 |
| L lateral occipital cortex | 0.0045 | 0.15 | 53 |
| L middle temporal gyrus | 0.0049 | 0.30 | 25 |
| R lingual gyrus | 0.0051 | 0.32 | 23 |
| R middle temporal gyrus | 0.0067 | 0.20 | 36 |
| R fusiform | 0.0068 | 0.23 | 31 |
| L precuneus | 0.0081 | 0.11 | 63 |
| L precentral gyrus | 0.0082 | 0.08 | 92 |
| R postcentral gyrus | 0.0089 | 0.15 | 45 |
| R pars orbitalis | 0.012 | 0.48 | 12 |
| L pars opercularis | 0.014 | 0.13 | 47 |
| L inferior parietal cortex | 0.015 | 0.08 | 75 |
| L lingual gyrus | 0.018 | 0.17 | 33 |
| L posterior cingulate | 0.027 | 0.34 | 14 |
| L cuneus | 0.033 | 0.24 | 19 |
| R posterior cingulate | 0.036 | 0.40 | 11 |
| R cuneus | 0.036 | 0.23 | 19 |
| R superior temporal gyrus | 0.048 | 0.21 | 19 |
| R lateral orbitofrontal cortex | 0.048 | 0.12 | 32 |
| L lateral orbitofrontal cortex | 0.063 | 0.19 | 19 |
| L pars triangularis | 0.09 | 0.13 | 23 |
| R bank of the superior temporal sulcus | 0.11 | 0.20 | 14 |
| L caudal anterior cingulate | 0.19 | 0.09 | 21 |
| R pericalcarine | 0.21 | 0.17 | 11 |
| L pericalcarine | 0.32 | 0.05 | 23 |
| L bank of the superior temporal sulcus | 0.65 | 0.01 | 18 |
| L insula | 0.84 | 0.001 | 39 |

R^2^ = coefficient of determination*, n* = number of participants.

R = right hemisphere, L = left hemisphere.

**Table S3.** All regions-of-interest (ROI) evaluated in linear regression analyses for stop-signal PSC and BH PSC. *P* and R^2^ values from the linear regression analyses shown.

| **ROI** | ***P*-value** | **R^2^** | ***n*** |
| --- | --- | --- | --- |
| R inferior parietal cortex | 8.6 x 10^-9^ | 0.58 | 41 |
| R superior temporal gyrus | 1.8 x 10^-7^ | 0.38 | 60 |
| R supramarginal gyrus | 8.0 x 10^-7^ | 0.36 | 58 |
| L supramarginal gyrus | 1.6 x 10^-6^ | 0.42 | 45 |
| L inferior parietal cortex | 2.1 x 10^-6^ | 0.63 | 25 |
| L superior parietal cortex | 8.9 x 10^-6^ | 0.54 | 28 |
| L lateral occipital cortex | 1.6 x 10^-5^ | 0.48 | 31 |
| R superior frontal gyrus | 2.2 x 10^-5^ | 0.47 | 31 |
| R middle temporal gyrus | 3.4 x 10^-5^ | 0.36 | 41 |
| R rostral middle frontal gyrus | 6.0 x 10^-5^ | 0.38 | 37 |
| R precuneus gyrus | 1.6 x 10^-4^ | 0.47 | 25 |
| L rostral middle frontal gyrus | 0.0011 | 0.35 | 27 |
| R bank of the superior temporal sulcus | 0.0014 | 0.25 | 39 |
| L superior temporal gyrus | 0.0015 | 0.17 | 56 |
| R superior parietal cortex | 0.0022 | 0.29 | 30 |
| R pars opercularis | 0.0027 | 0.51 | 15 |
| L superior frontal gyrus | 0.0037 | 0.30 | 26 |
| R precentral gyrus | 0.0074 | 0.26 | 26 |
| L middle temporal gyrus | 0.011 | 0.30 | 21 |
| L caudal middle frontal gyrus | 0.013 | 0.45 | 13 |
| L posterior cingulate | 0.015 | 0.50 | 11 |
| L precuneus | 0.015 | 0.37 | 15 |
| L precentral gyrus | 0.020 | 0.22 | 24 |
| R caudal middle frontal gyrus | 0.027 | 0.27 | 18 |
| L postcentral gyrus | 0.033 | 0.21 | 22 |
| R lateral orbitofrontal cortex | 0.038 | 0.36 | 12 |
| R pars triangularis | 0.066 | 0.33 | 11 |
| L insula | 0.084 | 0.23 | 14 |
| L bank of the superior temporal sulcus | 0.13 | 0.15 | 17 |
| R insula | 0.15 | 0.12 | 18 |
| L pars opercularis | 0.48 | 0.07 | 10 |
| R postcentral | 0.58 | 0.02 | 15 |
| R inferior temporal gyrus | 0.78 | 0.006 | 14 |
| R lateral occipital cortex | 0.91 | 0.0005 | 26 |

R^2^ = coefficient of determination*, n* = number of participants.

R = right hemisphere, L = left hemisphere.

**Table S4.** All regions-of-interest (ROI) evaluated in linear regression analyses for task-switch PSC and BH PSC. *P* and R^2^ values from the linear regression analyses shown.

| **ROI** | ***P*-value** | **R^2^** | ***n*** |
| --- | --- | --- | --- |
| L superior frontal gyrus | 1.8 x 10^-4^ | 0.62 | 17 |
| L superior parietal cortex | 5.9 x 10^-4^ | 0.31 | 34 |
| R pars opercularis | 0.0011 | 0.67 | 12 |
| R lingual gyrus | 0.0020 | 0.31 | 28 |
| R precuneus | 0.0025 | 0.45 | 18 |
| L inferior parietal cortex | 0.0044 | 0.31 | 24 |
| R lateral occipital cortex | 0.0048 | 0.53 | 13 |
| L lingual | 0.0054 | 0.22 | 34 |
| L precentral gyrus | 0.010 | 0.23 | 28 |
| L precuneus | 0.012 | 0.34 | 18 |
| L supramarginal gyrus | 0.012 | 0.25 | 24 |
| R supramarginal gyrus | 0.037 | 0.37 | 12 |
| L pericalarine | 0.050 | 0.21 | 19 |
| R superior parietal cortex | 0.060 | 0.17 | 22 |
| L rostral middle frontal gyrus | 0.078 | 0.14 | 24 |
| R precentral gyrus | 0.090 | 0.22 | 14 |
| L fusiform | 0.14 | 0.13 | 18 |
| R inferior parietal cortex | 0.21 | 0.10 | 17 |
| L postcentral gyrus | 0.30 | 0.06 | 19 |
| L caudal middle frontal gyrus | 0.39 | 0.06 | 15 |
| R superior frontal gyrus | 0.43 | 0.08 | 10 |
| L lateral occipital cortex | 0.77 | 0.004 | 23 |
| R rostral middle frontal gyrus | 0.85 | 0.003 | 16 |
| R pericalcarine | 0.94 | 0.0006 | 11 |

R^2^ = coefficient of determination*, n* = number of participants.

R = right hemisphere, L = left hemisphere.

**
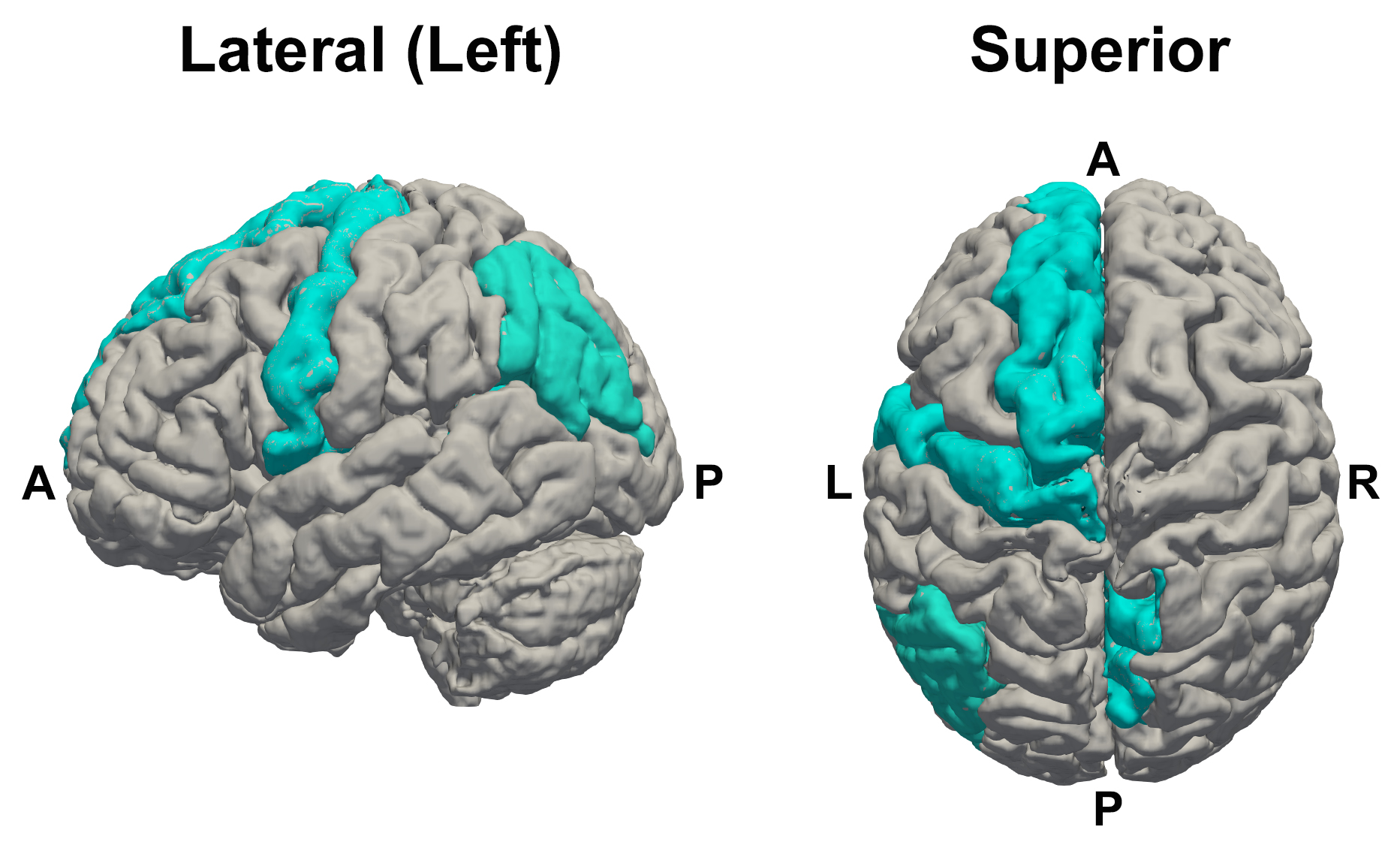
**

**Figure S1.** Lateral view of the left hemisphere (left) and superior (right) views of the ROIs showing a significant linear relationship between task-based fMRI activation and BH BOLD responses in all 3 cognitive paradigms. Left inferior parietal cortex, left precentral gyrus, left superior frontal gyrus, left supramarginal gyrus and right precuneus shown in cyan. A = anterior, P = posterior, L = left, R = right.

**Table S5.** All regions-of-interest (ROI) evaluated in linear regression analyses for ANT-R PSC and CVR. *P* and R^2^ values from the linear regression analyses shown.

| **ROI** | ***P*-value** | **R^2^** | ***n*** |
| --- | --- | --- | --- |
| R paracentral gyrus | 2.8 x 10^-4^ | 0.71 | 13 |
| R cuneus | 0.0010 | 0.64 | 13 |
| R pericalcarine | 0.0042 | 0.51 | 14 |
| L paracentral lobule | 0.022 | 0.42 | 12 |
| R inferior parietal cortex | 0.042 | 0.28 | 15 |
| L precuneus | 0.064 | 0.30 | 12 |
| L cuneus | 0.067 | 0.23 | 15 |
| R caudal anterior cingulate | 0.079 | 0.26 | 13 |
| L superior frontal gyrus | 0.093 | 0.20 | 15 |
| L middle temporal gyrus | 0.097 | 0.25 | 12 |
| R insula | 0.12 | 0.21 | 13 |
| L inferior parietal cortex | 0.12 | 0.18 | 15 |
| R bank of the superior temporal sulcus | 0.16 | 0.23 | 11 |
| L insula | 0.14 | 0.20 | 12 |
| L pars triangularis | 0.15 | 0.20 | 12 |
| R precuneus | 0.18 | 0.17 | 12 |
| R superior frontal gyrus | 0.19 | 0.13 | 15 |
| R superior parietal cortex | 0.20 | 0.12 | 15 |
| R superior temporal gyrus | 0.25 | 0.11 | 14 |
| L postcentral gyrus | 0.30 | 0.08 | 15 |
| L caudal anterior cingulate | 0.32 | 0.11 | 11 |
| R superior temporal gyrus | 0.32 | 0.09 | 13 |
| R precentral gyrus | 0.35 | 0.07 | 15 |
| R caudal middle frontal gyrus | 0.36 | 0.07 | 15 |
| L supramarginal gyrus | 0.39 | 0.06 | 15 |
| R middle temporal gyrus | 0.40 | 0.07 | 13 |
| R posterior cingulate | 0.50 | 0.04 | 14 |
| R supramarginal gyrus | 0.51 | 0.03 | 15 |
| R inferior temporal gyrus | 0.53 | 0.03 | 15 |
| R pars triangularis | 0.55 | 0.04 | 12 |
| R postcentral gyrus | 0.55 | 0.03 | 15 |
| R pars opercularis | 0.57 | 0.03 | 14 |
| L lateral occipital cortex | 0.66 | 0.02 | 15 |
| L inferior temporal gyrus | 0.69 | 0.02 | 13 |
| R rostral middle frontal gyrus | 0.70 | 0.01 | 15 |
| L posterior cingulate | 0.72 | 0.01 | 12 |
| L bank of the superior temporal sulcus | 0.72 | 0.01 | 12 |
| R lingual gyrus | 0.74 | 0.009 | 15 |
| L rostral middle frontal gyrus | 0.74 | 0.009 | 14 |
| L lingual gyrus | 0.76 | 0.009 | 13 |
| L pericalcarine | 0.77 | 0.008 | 13 |
| L lateral orbitofrontal cortex | 0.81 | 0.005 | 13 |
| R fusiform | 0.82 | 0.005 | 13 |
| L precentral gyrus | 0.82 | 0.004 | 15 |
| R lateral occipital cortex | 0.84 | 0.003 | 15 |
| L fusiform | 0.86 | 0.003 | 13 |
| L transverse temporal cortex | 0.89 | 0.002 | 12 |
| L caudal middle frontal gyrus | 0.93 | 0.0006 | 15 |
| L pars opercularis | 0.94 | 0.0006 | 12 |
| L superior parietal cortex | 0.99 | 0.00002 | 15 |

R^2^ = coefficient of determination*, n* = number of participants.

R = right hemisphere, L = left hemisphere.

**Figure S2.** Scatterplot showing the task-based SCAP percent signal change (%SC) vs. breath-hold BOLD percent signal change for the left insula in participants aged 40+ years (in blue) and participants aged 21 to 29 years (in magenta) at the time of testing. This plot shows that the older group had a non-significant negative relationship between breath-hold and task-based BOLD activation for this task (P > 0.05), while the younger group showed a significant (P = 0.05) positive relationship. These disparate age-dependent relationships may explain why the null hypothesis was retained for the left insula in the main analysis, where all data were averaged over age groups.
